# Scorpion Biodiversity and Interslope Divergence at “Evolution Canyon”, Lower Nahal Oren Microsite, Mt. Carmel, Israel

**DOI:** 10.1371/journal.pone.0005214

**Published:** 2009-04-09

**Authors:** Shmuel Raz, Sion Retzkin, Tomáš Pavlíček, Adam Hoffman, Hagay Kimchi, Dan Zehavi, Avigdor Beiles, Eviatar Nevo

**Affiliations:** 1 Department of Evolutionary and Environmental Biology, University of Haifa, Haifa, Israel; 2 Institute of Evolution, International Graduate Center of Evolution, University of Haifa, Haifa, Israel; 3 Institute of Evolution, Evolution Youth Cadets, University of Haifa, Haifa, Israel; University of Texas Arlington, United States of America

## Abstract

**Background:**

Local natural laboratories, designated by us as the “Evolution Canyon” model, are excellent tools to study regional and global ecological dynamics across life. They present abiotic and biotic contrasts locally, permitting the pursuit of observations and experiments across diverse taxa sharing sharp microecological subdivisions. Higher solar radiation received by the “African savannah-like” south-facing slopes (AS) in canyons north of the equator than by the opposite “European maquis-like” north-facing slopes (ES) is associated with higher abiotic stress. Scorpions are a suitable taxon to study interslope biodiversity differences, associated with the differences in abiotic factors (climate, drought), due to their ability to adapt to dry environments.

**Methodology/Principal Findings:**

Scorpions were studied by the turning stone method and by UV light methods. The pattern observed in scorpions was contrasted with similar patterns in several other taxa at the same place. As expected, the AS proved to be significantly more speciose regarding scorpions, paralleling the interslope patterns in taxa such as lizards and snakes, butterflies (Rhopalocera), beetles (families Tenebrionidae, Dermestidae, Chrysomelidae), and grasshoppers (Orthoptera).

**Conclusions/Significance:**

Our results support an earlier conclusion stating that the homogenizing effects of migration and stochasticity are not able to eliminate the interslope intra- and interspecific differences in biodiversity despite an interslope distance of only 100 m at the “EC” valley bottom. In our opinion, the interslope microclimate selection, driven mainly by differences in insolance, could be the primary factor responsible for the observed interslope pattern.

## Introduction

Scorpions inhabit all terrestrial habitats with the exception of tundra, high-latitude taiga, and some high-elevation mountain tops and their spatial distribution is influenced, to a large extent, by temperature and precipitation [1]. Interestingly, their taxonomic richness is maximal in subtropical areas (23–38° latitude), decreasing toward the poles and the equator [1]. In contrast to many other animal taxa that are most diverse in humid tropics, the most diverse scorpion communities occur in xeric areas [2], [3], probably due to their remarkable ability to adapt, such as with their resistance to high temperatures [4] and ability to conserve water for prolonged periods even at low air humidity [5]. As a consequence of the scorpion's ability to live in dry environments, the small arid and mesic Israeli habitats were inhabited by three out of the 16 recognized scorpion families [6]: Buthidae, Scorpionidae, and Diplocentridae. In Israel, these families comprised nine genera, 19 species and subspecies, out of about 1,200 known species (1.66%), which belong to three faunal elements, Saharo-Sindian (most species), Mediterranean, and Central Asian [7], [8].

The objective of the present study was to study the local pattern of distribution of biodiversity of scorpions at the Mediterranean microsite designated “Evolution Canyon”, lower Nahal Oren, Mount Carmel, Israel (EC). This study is part of a multidisciplinary research program to highlight the determinants of biodiversity distribution from cyanobacteria to mammals (summarized in 9–12).

## Materials and Methods

### Site description

**Figure 1:**
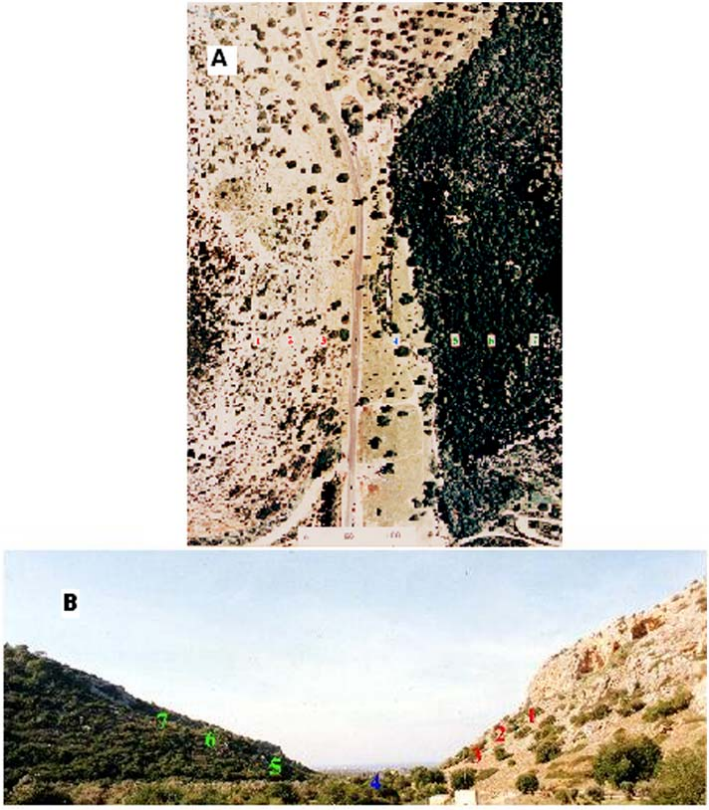
“Evolution Canyon”, Lower Nahal Oren, Mount Carmel, Israel. A.Aerial View, B. Cross section.

Evolution Canyon I (EC, Fig. 1) - located at the lower part of Nahal Oren (32°43′N; 34°58′E) – is a deeply incised summer-dry valley draining Mt. Carmel into the Mediterranean Sea in an east-west direction. The valley bottom is 40 m asl., the “African savannah–like” south-facing slope (AS) dips 20°–40°, and the “European maquis–like” north-facing slope (ES) dips 20°–30°. Geology (presumably 3–5 million years, an old Plio-Pleistocene canyon cut in the Upper Cenomanian Limestone [13], [9]), regional Mediterranean climate (mean annual rainfall ca. 600 mm, potential evapotranspiration 1700 mm, and mean August and January temperature 28°C and 13°C, respectively: [14]), and soil type (terra rosa [9]) are the same on both slopes. Interslope distance is 100 m at the bottom and 400 m at the top; The “African savannah–like” south-facing slope AS and the “European maquis–like” north-facing slope (ES) are 120 m and 180 m long, respectively (Fig. 1). The AS is significantly warmer, less humid, more illuminated, and microclimatically more fluctuating than the opposite ES due to differences in geographic orientation [15]. The life form analysis clearly illustrates the dramatic interslope microclimate differences. The hot, xeric AS is covered by the Mediterranean savannoid formation of *Ceratonia siliqua-Pistacia lentiscus* and the ES – by the dense maquis of *Quercus calliprinos-Pistacia palaestina* [16]. The plant cover varies from 35% on the AS to 150% on the ES [16].

#### Sampling Stations

The AS has three sampling stations - AS1, AS2, and AS3- at elevations 120 m, 90 m, and 60 m above sea level (figure 1) The ES has three sampling stations as well - ES5, ES6, and ES7 - at 60 m, 90 m, and 120 m above sea level. The area of each station is 1000 meter^2^ (10 m×100 m). All these stations lie perpendicular to the valley bottom.

### Sampling and recording

Sampling at “EC” was conducted during the following periods: (i) from November 1993 till October 1994 (19.5 days - 78 person hours), (ii) from February 2003 till December 2003 (21 days - 84 person hours), and (iii) from July till October 2007 (3.5 days - 14 person hours). The effort invested in scorpion sampling was the same at each sampling station on each recording day. The AS and ES stations were sampled on the same days (using the turning stone method) or on the same nights (UV-light method). Scorpions were sampled by the turning stone method during the first two collection periods and by the UV-light method (using AB-406U lamp) in the third collection period.

### Statistics

Since the data sets were not parametrically distributed, we used the factor analysis based on the Spearman correlation matrix to estimate the number of factors needed to explain inter-station variability in species richness. To test the significance of the interslope differences, we used the following model (abbreviated Interslope Difference or “ID” model) based on the Median test [17], [18]. According to this model, the interslope difference is significant (*p* = 0.05) if the species richness of any taxa is higher on the three stations of one slope in comparison to species richness of the same taxon on the three stations of the opposite slope. The same logic applies to the interslope comparisons of abundance. We did not consider the rank of stations on the same slope because we had no prediction for it. This procedure is rather conservative (*e.g.*, in comparison with the Binomial test) but, in our opinion, more realistic because, at least partly, it eliminates collection bias. In a few cases, we used the Binomial test (http://home.clara.net/sisa/binomial.htm). To perform the statistical analyses we used statistical programs Statistica for Windows, Ver. 6.0 (Tulsa OK. Stevenson RJ, 1996) and KyPlot, Ver. 2.0 (Yoshiyota K,1997)

### Fluctuating habitat asymmetry in scorpions and other taxa

We tested for fluctuating asymmetry (FA), which is small, non-directional, and normally distributed deviations from perfect symmetry [19], in 193 species belonging to seven groups of taxa more speciose on the AS [Review in Nevo], [9]–[12]. The fluctuating asymmetry for each species was calculated by subtracting the number of specimens that were found on the AS from those on the ES in absolute value and were normalized to the total number of specimens on both slopes 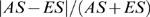 [20], [21]. The directional asymmetry was calculated as well 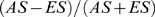. In order to test for directional asymmetry, we used one sample two tailed t-test of 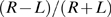 against zero [22]–[23]; separately for each one of the different taxa groups. In order to compare the FA level among the groups, we used the non-parametric Kruskal-Wallis test. We additionally tested for normality of the signed asymmetry using the Shapiro-Wilk test.

## Results

We analysed species richness and abundance independently because species richness per station and abundance per station (Table 1) were not significantly correlated (Kendal Rank Correlation, Tau = 0.27, *p* = 0.42).

**Table 1:** Scorpion species and their abundance recorded at “EC”.

Species	AS1	AS2	AS3	ES5	ES6	ES7	AS/ES	P_(B)_/P_(ID)_
	t_1_+t_2_+t_3_	t_1_+t_2_+t_3_	t_1_+t_2_+t_3_	t_1_+t_2_+t_3_	t_1_+t_2_+t_3_	t_1_+t_2_+t_3_		
1. *A. crassicauda*	0+0+0	0+0+0	1+1+0	0+0+0	0+0+0	0+0+0	2/0	?/Ns
2. *H. judaicus*	0+0+0	3+5+0	2+0+2	0+0+0	0+0+0	0+0+0	Dec-00	**/Ns
3. *C. werneri schmiedeknechti*	3+1+1	3+4+1	11+1+1	0+7+1	0+2+0	1+3+0	26/14	Ns/Ns
4. *C. carmelitis*	0+1+0	0+1+0	0+1+0	0+0+0	0+0+0	0+2+0	3/2	Ns/Ns
5. *S. maurus fuscus*	1+3+0	7+2+1	20+2+0	1+5+0	1+0+0	0+0+0	36/7	**/Ns
6. *N. hierichonticus*	1+1+0	2+5+0	4+18+1	6+15+0	13+14+0	0+13+0	32/61	**/*
Total abundance	5+6+1 = 12	15+17+2 = 34	38+23+4 = 65	7+27+1 = 35	14+16+0 = 30	1+18+0 = 19	111/84	*/Ns
Species richness/total no. of species	3+4+1/4	4+5+2/5	5+5+3/6	2+3+1/3	2+2+0/3	1+3+0/3	4-Jun	Ns/*

P_(B)_ - Binomial test (expected proportion = 0.5), P_(ID)_ – ID model, t_1_ = 1993/1994, t_2_ = 2003, t_3_ = 2007. Ns = insignificant, *p≤0.05, **p≤0.01, ? – no power of the test.

### Species richness

All together six scorpion species were recorded at EC (Text S1, Table 1); out of these, five species were recorded during the first sampling season, six species during the second collection season, and four by the UV method (Table 1). There were no inter-station significant differences in the recorded species richness between the first (2.83±2.17) and second (3.67±1.47) collection periods. We excluded the third collection period (2007) from the comparison with previous observations because a different recording method was used and it contributed only 7.8% of the invested time compared with 44%, 47.4% (2003), and 44% (1993/1994).

*C. carmelitis* was recorded exclusively during the 2003 recording season. All six recorded scorpion species were present on the AS, and four of them were also found on the ES (Table 1). The interslope difference in species richness was significant according to the ID model because each of the AS stations harboured more species than any of the ES stations (Table 1). Two species (*A. crassicauda*, *H. judaicus*) were AS-unique.

### Abundance

A total of 195 specimens were recorded at “EC” (Table 1). Rejection of the null hypothesis, which predicted a homogeneous distribution of all species on both slopes by the chi-square test (X^2^ = 5, *p* = 0.0003), permitted us to conduct individual tests. The two alternative null hypotheses: (i) equal binomial probability on each slope and (ii) binomial probability based on the slope total, were rejected (Table 1). The interslope difference in abundance did not reach statistical significance. However, *Compsobuthus werneri schmiedeknechti* showed significantly (by both, binomial test and ID model: Table 1) higher abundance on the AS than on the ES and *Nebo hierichonticus* on the ES than on the AS.

During the first sampling period (1993–1994) and the second one (2003), four species (*H. judaicus*, *S. maurus*, *A. crassicauda*, and *N. hierochunticus*) out of six displayed the same pattern of interslope distribution (Table 1). One species, *Compsobuthus werneri schmiedeknechti*, displayed a contradiction pattern of distribution between the first two periods of collection (17 specimens on the AS and 1 specimen on the ES during the first period vs. 6 specimens on the AS and 12 specimens on the ES during the second period). The third period of collection contributed only eight specimens in total, therefore, it wasn't included in this comparison.

Comparison of the recorded abundance of this species in 2003 (five recorded specimens) and 1993/1994 (zero recorded specimens) indicates significant inter-sample difference (Binomial test, *p* = 0.03).

### Interslope distribution of scorpions and other phylogenetic groups

**Figure 2:**
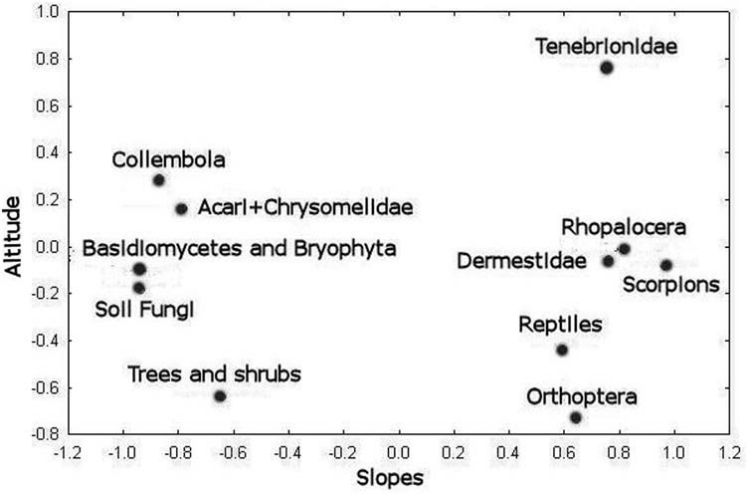
Distribution of taxa and ecological groups in “Evolution Canyon”.

Factor analysis, using the Spearman correlation matrix (based on ranking species richness according to stations) of the groups listed in Table 2 [22]–[27], indicates that Scorpions are classified together with taxa more speciose on the AS than the ES such as reptiles, butterflies (Rhopalocera), darkling beetles (Tenebrionidae), skin beetles (Dermestidae), and grasshoppers (Orthoptera) (positive factor 1 in Fig. 2) and in the opposite direction of taxa more speciose on the ES than the AS such as springtails (Collembola), soil fungi, basidiomycete fungi (Basidiomycetes), mosses (Bryophyta), and trees and shrubs (negative factor 1 in Fig 2 and Table 2). The most important is factor 1 (eigenvalue = 7), which explains 64% of variance and differentiates between slopes. The role of factor 2 (eigenvalue = 1.88) associated with slope altitude is less important since it explains only 17% of the total variance.

**Table 2:** Ranking of station at “EC” according to increasing species richness in different phylogenetic groups.

Group	AS1	AS2	AS3	ES5	ES6	ES7	Factor 1	Factor 2
Scorpions*	4	5	6	3	3	3	0.96	−0.08
Mites*	3	2	1	5	4	6	−0.78	0.12
Reptiles^(22)^ *	6	5	4	1	2	3	0.59	−0.44
Soil fungi^(23,24)^ *	2	3	1	5	6	4	−0.93	−0.18
Rhopalocera^(25)^ *	4.5	6	4.5	1.5	1.5	3	0.82	−0.01
Orthoptera^(26)^	2.5	5	6	2.5	2.5	2.5	0.65	−0.74
Dermestidae^(27)^	4	4	6	4	1	2	−0.76	0.05
Chrysomelidae^(27)^ *	4	5	6	2	3	1	−0.78	0.12
Collembola^6^ *	2	3	1	6	4.5	4.5	−0.87	0.29
Basidiomycetes^(24)^ *+Bryophyta^(24)^ *	1.5	3	1.5	6	5	4	−0.93	−0.10
Tenebrionidae^(27)^ *	5	4	6	2	2	2	0.75	0.76
Trees+shrubs^16^ *	1	2.5	2.5	5	6	4	−0.64	−0.64
Eigenvalue							7.013	1.88
Proportion of the total (%)							63.8	17.1

* significant interslope difference according to the ID model. Ranking of Basidiomycetes and Bryophyta was identical. Factor 1 and Factor 2 explain the estimated variability in the Spearman correlation matrix by means of factor analysis.

### Fluctuating habitat asymmetry in species abundance of scorpions and other taxa

The fluctuating habitat asymmetry (FHA) in taxa more speciose on the AS than the ES is shown in table 3. There were no significant differences among the studied groups (*p* = 0.168, Kruskal_Wallis test). Scorpions and skin beetles displayed the lowest FA indicating a low level of stress, which they encounter at “EC” in contrast to Chrysomelidae (leaf beetles) and Rhopalocera (butterflies). There is no correlation between species abundance and FA rank (Spearman Correlation coefficient = 0.036, *p* = 0.939), but groups with a higher number of species displayed higher FA value (Spearman Correlation coefficient = 0.829, *p* = 0.021). Six taxa displayed directional asymmetry, which indicates higher distribution on the AS and six taxa displayed abnormal distribution. The only group that displayed pure FA (normal distribution around the mean of zero) was the scorpions.

**Table 3:** Fluctuating and directional asymmetry in species abundance.

Group	Mean FA rank*	Number of species	P value of Directional asymmetry**	P value of Normality test***	Average number of specimens per species
Scorpions	62.58	6	0.072	0.513	32.50
Dermestidae	73.63	15	0.371	0.053	8.87
Reptiles	74.31	13	0.013	0.016	689.4
Orthoptera	79.17	15	0.001	0.003	29.90
Tenebrionidae	84.90	34	0.001	0.0009	10.26
Chrysomelidae	94.1	86	0.001	0.0009	10.09
Rhopalocera	104.33	24	0.0009	0.0009	100.79

* In order to compare the FA level among the groups, we used the non-parametric Kruskal-Wallis test.

** In order to test for directional asymmetry, we used one sample two tailed t-test of 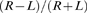 against zero, separately for each one of the different groups.

*** We tested for normality of the signed asymmetry using the Shapiro-Wilk test.

## Discussion

### Species richness

The six scorpion species recorded at “EC” represent 31.57% of scorpions known in Israel (19 species [7]). Our estimation of species richness at EC could be biased by using the turning stones method, which delivers a sample bias towards non-burrowing species [28] and due to the presence of species whose abundance was below the detection threshold of the used methods. As matter of fact, we could've missed the rare *Androctonus bicolour*, known also from Mt. Carmel [29]. The sampling bias towards non-burrowing species might be negligible because the three recorded non-burrowers (*H. judaicus*, *C. werneri schmiedeknechti*, and *C. Carmelites*) displayed 29% of the total abundance only. Even if the bias towards non-burrowing species is real, interslope comparisons can still be carried out on each species.

The first two recording seasons were similar, and no significant fluctuations were detected regarding recorded species richness. Only *C. carmelitis* was recorded exclusively in 2003. The appearances of significant inter-annual fluctuations in species composition were recorded in EC earlier, for example, in beetles and in fruit flies [27], [30], [31]. Some evidence indicates that inter-annual species presence-absence pattern might be described as a game of species to gain their presence against deteriorative fluctuations in environmental conditions at EC [32]. We do not expect that there was a serious under-estimation of scorpion species richness at EC because sampling by means of the UV method did not add additional scorpion species and the EC/Israel proportion in scorpion species richness is higher than that in many other studied invertebrate taxa. For example, the EC/Israel proportion was reported 23% in earthworms [33] scorpion species richness estimated at the AS than at the ES support the expectations, and it is similar to the scorpions' global distribution pattern with maximum species inhabiting subtropical areas (23–38° latitude), of the world [1]. Similar interslope patterns, showing higher species richness at AS than ES and locally mirroring the patterns of global distribution, have been reported in most other “heat-dependent” (terrestrial in [9]) taxa tested hitherto at EC such as Reptiles, Rhopalocera, Tenebrionidae, Dermestidae, Chrysomelidae, and Orthoptera [summarized in 9]–[12], [34]. In contrast to “heat-dependent” taxa the less speciose “humidity-dependent” taxa (e.g., fungi, woody plants, oribatid mites) are more speciose at ES [31]. This substantiated the hypothesis of higher biodiversity in the tropical savanna-like habitats of AS compared with the “temperate” ES [9]–[12].

### Faunal elements

The Mediterranean elements penetrating into steppe areas (*Hottentotta judaicus*, *Compsobuthus werneri schmiedeknechti*, *Compsobuthus carmelitis* and *Scorpio maurus fuscus*), occur only in the north and central parts of Israel. Five-out-of-six scorpion species found at “EC” only or largely on the AS represent the preference of scorpions to warm and xeric habitats [3], [4]. Likewise, their local distribution mimics the fact that their southern border of their geographic distribution is the Judean Mountains (*H. judaicus*), Gaza (*C. w. schmiedeknechti*), the steppic region south of Bet Shean (*S. m. fuscus*), or Sinai (*A. crassicauda*). An odd discovery of our study is the distribution of *N. hierichonticus*, which is a Saharo-Sindian scorpion known from Arabia, Sinai, and Negev deserts, which also ranges in mesic Mediterranean Israel. At “EC”, this scorpion ranges primarily on the mesic ES, rather than on the xeric AS.

Our study at “EC” sheds some light on the differential ecophysiology of *Scorpio maurus fuscus* and *Nebo hierichonticus*. Both species also occur in the mesic areas of the Mediterranean regions of northern Israel. However, our study clearly showed microhabitat divergence between these two scorpions. Very clearly, *S. m. fuscus* significantly prefers the xeric AS of the “EC”, whereas *N. hierichonticus* prefers the mesic ES. This seems odd in view of the fact that the latter ranges in the xeric Sinai and Negev deserts, whereas the former is confined to mesic Mediterranean and steppic Jordan Valley regions in Israel. Possibly, this can be a case of competitive exclusion. This idea is supported by experimental evidence showing that *S. m fuscus* and *N. hierichonticus* displayed a higher rate of water loss under the same extreme conditions in comparison with *Hottentotta judaicus* but that *S. m. fuscus* showed a broader and higher temperature preference range than *N. hierichonticus* [35]–[37]. If correct, then the stenothermic *N. hierichonticus* over competes eurythermic *S. m. fuscus* on the more mesic ES and on the climatically more stressful AS the outcome of the competitive exclusion between both species is reversed.

Could it be that the competitive exclusion could be followed by ecological exclusion leading to ecophysiological and possibly systematic and evolutionary divergence between xeric and mesic populations in *N. hierichonticus* and *S. m. fuscus* in northern Israel? The resolution of this evolutionary dilemma calls for an in-depth molecular-genetic analysis that might highlight the biological significance of the observed peculiar distributions at “EC” in *N. hierichonticus* and *S. m. fuscus*. Testing the temperature preference of xeric populations might be of great interest. However, the goal of this paper was not to analyze these peculiarities. In view of our microscale results, a thorough genetic, physiological, and behavioral analysis is required to highlight the evolutionary history as well as adaptive and possible speciational patterns of scorpions in northern Israel.

### Conclusions

Differential distributional patterns in species richness and species abundance along the climatic transect of a few hundred meters (the geology is identical on both slopes) reveals ecological (climatic) selection strong enough to override the mixing effects of migration and stochasticity. The ecological selection caused by higher insolance leads to the higher ecological heterogeneity of the savanna-type ecosystem on the AS that accommodates more species of heat-dependent taxa. In other words, the species richness differentiation pattern observed on a microscale resemble the one observed generally on a macroscale in tropical regions [9]–[12].

## Supporting Information

Text S1 Studied species
(0.03 MB DOC)
