# Supplementary material for: Scorpion Biodiversity and Interslope Divergence at “Evolution Canyon”, Lower Nahal Oren Microsite, Mt. Carmel, Israel
Source: PLoS One. 2009 Apr 9;4(4):e5214. doi: 10.1371/journal.pone.0005214 (PMC2664475; doi:10.1371/journal.pone.0005214)
Supplement: Text S1 — Studied species (0.03 MB DOC) [file pone.0005214.s001.doc]

Appendix S1 – Studied species

*Hottentotta judaicus* (Simon, 1872) belongs to the largest cosmopolitan scorpion family, Buthidae, with 44 genera and 600 species. It represents a genus involving 20 species ranging across Africa and Asia from the Near East to Iran and India. *H. judaicus*, the only species of the genus in Israel, apparently ranges from Turkey through Syria (but certainly in Lebanon and Jordan) to northern and central Israel where it is common in the Mediterranean zone from the north (including the rift valley from Tiberias northward) to the Judean Mountains, south of Hebron. It lives under the bark of trees, in loosely built stone walls, and at “Evolution Canyon” under small and large stones.

*Compsobuthus werrneri schmiedeknechti* (Vachon, 1949) (Buthidae) belongs to a genus with 11 species ranging in northern Africa and in Asia from Turkey through Iran to India. *C. w. schmiedeknechti* ranges in Lebanon and Israel from the north to the southern Judea foothills and Gaza. It is common and found on various soil types. It lives in soil and rock crevices, under tree bark, and at “Evolution Canyon" under small and large stones.

*Compsobuthus carmelitis* (Levy, Amitai & Shulov, 1973) (Buthidae) belongs to a genus with 11 species ranging in North Africa and in Asia from Turkey through Iran to India. *C*. *carmelitis* were found only in few localities in the western Upper Galilee, Mount Carmel, and from Arbel in the eastern Lower Galilee. *C*. *carmelitis* is very rare. It is found on terra rossa, probably in slightly humid localities, under stones and at “Evolution Canyon” in cracks between small rocks.

*Androctonus crassicauda* (Olivier, 1807) (Buthidae) represents the central Asian element. It ranges in the Caucasus, Kurdistan, south Turkey (southern Anatolia only), Iran, Iraq, Syria, Jordan, Arabia, and Sinai reaching its western border of distribution in Israel. It is found throughout Israel and central and south Sinai, including Arava and Eilat, but not in sand dunes. The genus ranges from India to Iran and the Near East, and in Africa from Egypt and Sudan to Senegal. It is not common and is found in mountainous areas on various soil types. It was found at “Evolution Canyon” under large stones.

*Scorpio maurus fuscus* (Ehrenberg, 1829) belongs to the family Scorpionidae, including hundreds of species ranging in Africa, Asia, and Australia. The species *S. maurus* ranges in North Africa and in the Near East; it reaches Iran and Arabia. The two Israeli subspecies are apparently two species hybridizing around Lahav. *S. m fuscus* ranges from southeast Turkey through Syria and Lebanon to Israel, where it is found from the north to the Judean Mountains and along the xeric Jordan Valley to about 40 km south of Beth Shean. It is fairly common on various soil types. At “Evolution Canyon” it was found under stones usually as a burrowing form. Significantly, this species, which does penetrate in the xeric Jordan Valley in Israel, is primarily found on the south-facing slope.

*Nebo hierichonticus* (Simon, 1872) belongs to a relatively small family, the Diplocentridae, with only several dozen neotropical species. In our region the subfamily Nebinae involves two genera, and the genus Nebo involves only one species, *N. hierichonticus*; it ranges from Arabia reaching the Persian Gulf to northern Egypt through Sinai and Jordan. In Israel it ranges from the north to the southern Negev. It is the largest Israeli scorpion and is found on terra rosa, basalt, and rendzina soils and in stony deserts. At “EC” it was found under large stones, rock crevices and rodent burrows, although elsewhere else it digs burrows. All distributional records are from Levy and Amitai (1980).
